# Association of Alternative Payment and Delivery Models With Outcomes for Mental Health and Substance Use Disorders: A Systematic Review

**DOI:** 10.1001/jamanetworkopen.2020.7401

**Published:** 2020-07-23

**Authors:** Andrew D. Carlo, Nicole M. Benson, Frances Chu, Alisa B. Busch

**Affiliations:** 1 Department of Psychiatry and Behavioral Sciences, University of Washington School of Medicine, Seattle; 2 McLean Hospital, Harvard Medical School, Boston, Massachusetts; 3 University of Washington School of Nursing, Seattle; 4 Department of Health Care Policy, McLean Hospital, Harvard Medical School, Boston, Massachusetts

## Abstract

**Importance:**

Health care spending in the United States continues to grow. Mental health and substance use disorders (MH/SUDs) are prevalent and associated with worse health outcomes and higher health care spending; alternative payment and delivery models (APMs) have the potential to facilitate higher quality, integrated, and more cost-effective MH/SUD care.

**Objective:**

To systematically review and summarize the published literature on populations and MH/SUD conditions examined by APM evaluations and the associations of APMs with MH/SUD outcomes.

**Evidence Review:**

A literature search of MEDLINE, PsychInfo, Scopus, and Business Source was conducted from January 1, 1997, to May 17, 2019, for publications examining APMs for MH/SUD services, assessing at least 1 MH/SUD outcome, and having a comparison group. A total of 27 articles met these criteria, and each was classified according to the Health Care Payment Learning and Action Network’s APM framework. Strength of evidence was graded using a modified Oxford Centre for Evidence-Based Medicine framework.

**Findings:**

The 27 included articles evaluated 17 APM implementations that spanned 3 Health Care Payment Learning and Action Network categories and 6 subcategories, with no single category predominating the literature. APMs varied with regard to their assessed outcomes, funding sources, target populations, and diagnostic focuses. The APMs were primarily evaluated on their associations with process-of-care measures (15 [88.2%]), followed by utilization (11 [64.7%]), spending (9 [52.9%]), and clinical outcomes (5 [29.4%]). Medicaid and publicly funded SUD programs were most common, with each representing 7 APMs (41.2%). Most APMs focused on adults (11 [64.7%]), while fewer (2 [11.8%]) targeted children or adolescents. More than half of the APMs (9 [52.9%]) targeted populations with SUD, while 4 (23.5%) targeted MH populations, and the rest targeted MH/SUD broadly defined. APMs were most commonly associated with improvements in MH/SUD process-of-care outcomes (12 of 15 [80.0%]), although they were also associated with lower spending (4 of 8 [50.0%]) and utilization (5 of 11 [45.5%]) outcomes, suggesting gains in value from APMs. However, clinical outcomes were rarely measured (5 APMs [29.4%]). A total of 8 APMs (47.1%) assessed for gaming (ie, falsification of outcomes because of APM incentives) and adverse selection, with 1 (12.5%) showing evidence of gaming and 3 (37.5%) showing evidence of adverse selection. Other than those assessing accountable care organizations, few studies included qualitative evaluations.

**Conclusions and Relevance:**

In this study, APMs were associated with improvements in process-of-care outcomes, reductions in MH/SUD utilization, and decreases in spending. However, these findings cannot fully substitute for assessments of clinical outcomes, which have rarely been evaluated in this context. Additionally, this systematic review identified some noteworthy evidence for gaming and adverse selection, although these outcomes have not always been duly measured or analyzed. Future research is needed to better understand the varied qualitative experiences across APMs, their successful components, and their associations with clinical outcomes among diverse populations and settings.

## Key Points

**Question:**

Are alternative health care payment and delivery models (APMs) associated with changes in service delivery or outcomes for mental health and/or substance use disorders (MH/SUDs) in the United States?

**Findings:**

This systematic review included 27 articles on 17 APM implementations in MH/SUD care. Some specific APMs (eg, pay-for-performance) have been associated with improved MH/SUD outcomes, while others (eg, APMs with shared savings) have not; broadly, clinical outcome data are lacking in evaluations of APMs.

**Meaning:**

This systematic review identified some evidence for APM effectiveness in MH/SUD care; further research is needed to identify successful program components and associations with clinical outcomes.

## Teaser

This systematic review summarizes the published literature on populations and mental health and/or substance use disorder conditions examined by alternative payment and delivery models as well as the associations of these models with mental health and/or substance use disorder outcomes.

## Introduction

The continued growth in US health care spending^1,2^ and persistent suboptimal population health outcomes^3^ have spurred increasing interest by payers to tie clinician reimbursement to quality and value^4^ through alternative payment models (APMs). According to the Centers for Medicare & Medicaid Services (CMS), APMs provide financial incentives to encourage high-quality, cost-efficient care and can apply to a specific clinical condition, care episode, or population.^5^ APMs are heterogeneous and can be divided into groups based on the type of payment (eg, fee-for-service or population-level) and category of financial risk to the clinician or health care organization (ie, none, penalties, bonuses, or both). Given the potential of these models to shift the focus of care from volume to value, APMs have been the subject of several initiatives on the federal, state, and payer levels since the passage of the Patient Protection and Affordable Care Act in 2010.^6,7,8^

Mental health and substance use disorders (MH/SUDs), also known as behavioral health disorders, commonly co-occur and are associated with total health care spending that is 2 to 3 times higher than the national average.^9^ APMs have the potential to facilitate more efficient, comprehensive, and team-based care^10^ for this patient population by effectively aligning incentives at the patient, clinician, and system levels.^11^ Although MH/SUDs have rarely been prioritized in contemporary APM implementations,^12^ a body of work is now emerging.

To consolidate this literature, we conducted a systematic review with the primary aim of assessing the evidence base for the association of APMs with MH/SUD outcomes in the United States. We defined 5 outcome categories that are particularly relevant to MH/SUD care in the context of APMs, as follows: (1) process-of-care measures (to determine the extent to which clinical outcome–associated care processes are improved by APMs); (2) clinical outcomes; (3) spending; (4) utilization; and (5) adverse selection, patient dumping (ie, changes in the severity of patients treated in an APM context), or gaming (ie, falsification of outcomes because of APM incentives).

Additionally, we classified APMs according to the Health Care Payment Learning and Action Network (LAN) framework,^13,14^ which defines 4 categories (LAN 1 to LAN 4) that indicate movement along the continuum of integrated payment and delivery (Table 1). To our knowledge, this is the first such comprehensive review in the published academic literature. A 2017 Cochrane Systematic Review assessed APMs for general outpatient medical services worldwide^15^ and included 2 MH/SUD studies performed in the United States, both of which were identified in our query and excluded because they did not meet our APM definition (ie, payments were not tied to quality or value).^16,17^ Another 2017 systematic review restricted its search to pay-for-performance studies.^18^ Our investigation adds substantially to the literature by including a variety of payment models, conducting a multidimensional analysis, and organizing APMs for MH/SUDs and their respective evaluations by model type and evidence quality using common frameworks.

**Table 1.** The Health Care Payment LAN Framework^a^

LAN category	LAN subcategory	Description	Example
1, Fee-for-service, with no link to quality and value	NA	NA	Traditional fee-for-service payments to medical professionals and organizations
2, Fee-for-service with link to quality and value	A	Foundational payments for infrastructure and operations	Payments for health system infrastructure investments
	B	Pay-for-reporting	Bonuses for reporting data or penalties for failing to report data
	C	Pay-for-performance	Bonuses for high-quality performance
3, APMs built on fee-for-service architecture	A	APMs with shared savings	Shared savings with upside financial risk only (eg, Medicare Shared Savings Program ACOs)
	B	APMs with shared savings and downside risk	Episode-based payments for procedures and comprehensive payments with both upside and downside financial risk (eg, Medicare Pioneer ACOs)
	N	Risk-based payments not linked to quality	NA
4, Population-based payment	A	Condition-specific population-based payment	Per-member per-month payments or non–fee-for-service payments for specialty services treating populations defined by diagnosis or condition (eg, MH/SUDs); health organizations and payers share financial risk
	B	Comprehensive population-based payment	Non–fee-for-service global budgets or full/percentage of premium payments for defined populations not based on diagnosis or condition (eg, commercial payer ACO); health organizations and payers share financial risk
	C	Integrated finance and delivery system	Non–fee-for-service global budgets or full/percentage of premium payments in integrated systems (eg, Kaiser Permanente)
	N	Capitated payments not linked to quality	NA

Abbreviations: ACO, accountable care organization; APM, alternative payment and delivery model; LAN, Learning and Action Network; MH/SUD, mental health/substance use disorder; NA, not applicable.

^a^ Table adapted from 2 previous publications on the LAN framework.^13,14^

## Methods

After conducting a pilot search and systematic review on September 1, 2017, we performed the final search (with additional terms) on April 15, 2018. We updated the search on May 17, 2019. This investigation followed the Preferred Reporting Items for Systematic Reviews and Meta-analyses (PRISMA) reporting guideline.

The final literature search included MEDLINE, PsychInfo, Scopus, and Business Source databases and was restricted to studies between January 1, 1997, and May 17, 2019. Final search terms included 2 additions from the pilot search (eAppendix in the Supplement). We also manually searched reference lists of articles identified through the systematic review for other relevant citations. The primary focus of the literature review was comparative studies examining the associations of APMs with MH/SUD outcomes. For inclusion, articles had to describe studies from the United States, be written in English, examine an MH/SUD APM (according to the LAN framework^19^), assess a defined MH/SUD outcome, and have a comparison or control group.

For the first 100 study publications of the final literature search, 2 authors (A.D.C. and N.M.B.) reviewed titles and abstracts concurrently to identify articles for full review. After reaching concordance on article inclusions and exclusions, both authors independently reviewed half of the remaining article titles and abstracts. Articles that could not be excluded via this process were selected for full-text review. All discrepancies were resolved through email discussions and meetings. The senior author (A.B.B.) independently reviewed articles as needed.

Because multiple studies were often conducted on the same APM implementation, the data source for each article was identified and matches were noted. This allowed for analysis at the levels of APM implementation and article. APMs were categorized according to the LAN framework,^13,14^ with final designation being assigned by discussion and complete agreement among 3 authors (A.D.C., N.M.B., and A.B.B.). We excluded APMs in the LAN 3N and 4N categories because they lacked incentives or requirements regarding treatment quality or value.

Each article’s quality of study design was scored using a framework adapted from the Oxford Centre for Evidence-Based Medicine (OCEBM) (Table 2).^20,21^ The OCEBM framework alone is inadequate for evaluations of large, systems-level interventions used to inform health policy,^22^ scenarios in which randomized clinical trials are often infeasible. Evaluating these types of interventions requires additional design and analytic methods to mitigate threats to causal inference. Therefore, we augmented the OCEBM by dividing the level 3 rating (ie, natural experiments) into 3 groups based on the strength of the methods used to address threats of study bias or causal inference.^23^ In descending order of methodologic rigor, these were 3A, 3B, and 3C, with those in category 3C being at the highest risk of bias (Table 2).

**Table 2.** Quality Rating Scheme for Studies and Other Evidence Modified From the Oxford Centre for Evidence-Based Medicine Rating Framework^a^

Rating	Description
1	Properly powered and conducted randomized clinical trial; systematic review with meta-analysis
2	Well-designed controlled trial without randomization; prospective comparative cohort trial
3	Natural experiment, eg, case-control studies; retrospective cohort studies
3A	Comparison or control group or period, with regression analysis that includes robust observational study design methods, such as difference-in-differences analysis, instrumental variables, interrupted time series, or propensity scores, and the inclusion of a sensitivity analysis
3B	Comparison or control group or period with regression analysis but no sensitivity analysis
3C	Comparison or control group or period with no regression or sensitivity analysis
4	Case series with or without intervention; cross-sectional study
5	Opinion of respected authorities; case reports

^a^ Table adapted from the Oxford Centre for Evidence-Based Medicine Rating Framework and *JAMA Network Open*.^20,21^

### Statistical Analysis

No statistical analyses were conducted. Therefore, no software was used and no prespecified level of statistical significance was set

## Results

### Evidence Base

After removing duplicates, we identified 986 articles, 41 of which were classified as APMs. We excluded 13 because they were categorized LAN 4N^16,17,24,25,26,27,28,29,30,31,32,33,34^ and 1 because of concerns about data quality.^35^ This left 27 articles and 17 APM implementations in the final analysis. Details appear in the study flow diagram (eFigure in the Supplement). Each primary LAN APM category (ie, LAN 2 to LAN 4) was represented in the final sample, and 3 articles evaluated APMs in multiple LAN categories.^36,37,38^ No single primary LAN category predominated in the results (Table 3).^36,37,38,39,40,41,42,43,44,45,46,47,48,49,50,51,52,53,54,55,56,57,58,59,60,61,62^

**Table 3.** Results of Systematic Review Organized by the LAN Framework for APMs

LAN APM category^a^	APM implementation	Source	OCEBM quality rating^b^	Insurer type; age range	Outcome of focus
**2, Fee-for-service with link to quality and value**					
A, Foundational payments for infrastructure and operations	Sustaining Healthcare Across Integrated Primary Care Efforts Program	Ross et al,^39^ 2019	2	Medicare, Medicaid, and patients with dual eligibility; all ages	MH/SUDs
C, Pay-for-performance	Adolescent Community Reinforcement Approach	Garner et al,^40^ 2011^c^	1	Publicly funded SUD programs; child and adolescent	SUDs
		Garner et al,^41^ 2012	1		
		Garner et al,^42^ 2018	1		
		Lee et al,^43^ 2012	1		
	Spectrum Addiction Services	Shepard et al,^44^ 2006	3B	Medicaid and uninsured; adult	SUDs
	Outpatient psychosocial counseling treatment center in Maryland	Vandrey et al,^45^ 2011	3C	Publicly funded SUD programs; adult	SUDs
	Washington State Mental Health Integration Program	Unützer et al,^46^ 2012	3A	Medicaid; adult	MH
		Bao et al,^47^ 2017	3A		
	Connecticut’s Behavioral Health Partnership	Schmutte et al,^48^ 2019	3C	Medicaid; child and adolescent	MH/SUDs
**3, APMs built on fee-for-service architecture**^d^					
A, APMs with shared savings	Medicare Shared Savings Program Accountable Care Organizations	Busch et al,^36^ 2016	3A	Medicare; adult	MH
		Busch et al,^37^ 2017	3A		
	Maine Medicaid Accountable Communities Initiative	Beil et al,^38^ 2019	3A	Medicaid; all ages	MH/SUDs
	Vermont Medicaid Shared Savings Program	Beil et al,^38^ 2019	3A	Medicaid; all ages	MH/SUDs
B, APMs with shared saving and downside risk	Medicare Pioneer Accountable Care Organizations	Busch et al,^36^ 2016	3A	Medicare; adult	MH
		Busch et al,^37^ 2017	3A		
	Minnesota Integrated Health Partnerships Program	Beil et al,^38^ 2019	3A	Medicaid; all ages	MH/SUDs
**4, Population-based payment**					
A, Condition-specific population-based payment	Delaware Division of Substance Abuse and Mental Health–Outpatient Services APM	Stewart et al,^49^ 2013	3A	Publicly funded SUD programs; adult	SUDs
		McLellan et al,^50^ 2008	3C		
	Delaware Division of Substance Abuse and Mental Health–Detoxification Care Transition APM	Haley et al,^51^ 2011	3C	Publicly funded SUD programs; adult	SUDs
	Maine Addiction Treatment System, phase 1 of performance-based contracting	Commons et al,^52^ 1997	3B	Publicly funded SUD programs; adult	SUDs
		Lu,^53^ 1999	3B		
		Lu et al,^54^ 2003	3A		
		Lu and Ma,^55^ 2006	3A		
		Shen,^56^ 2003	3A		
	Maine Addiction Treatment System, phase 2 of performance-based contracting	Brucker and Stewart,^57^ 2011	3B	Publicly funded SUD programs; adult	SUDs
		Stewart et al,^58^ 2018	3A		
B, Comprehensive population-based payment	BCBSMA Alternative Quality Contract	Barry et al,^59^ 2015	3A	Commercial; adult	MH
		Stuart et al,^60^ 2017	3A		
		Donohue et al,^61^ 2018	3A		
	Oregon Coordinated Care Organizations	Rieckmann et al,^62^ 2018	3B	Medicaid; adult	SUDs

Abbreviations: APM, alternative payment and delivery model; BCBSMA, Blue Cross/Blue Shield of Massachusetts; LAN, Learning and Action Network; MH, mental health; OCEBM, Oxford Centre for Evidence-Based Medicine; SUD, substance use disorder.

^a^ Only LAN categories with examples appear in this table. All LAN categories appear in Table 1.

^b^ Adapted from the OCEBM Levels of Evidence by subdividing OCEBM category 3 into 3 levels (ie, A, B, and C) to account for substantial differences in research design and analysis quality between studies in this category. Descriptions of OCEBM levels appear in Table 2.

^c^ Garner et al^40^ primarily assessed the successful implementation of the Adolescent Community Reinforcement Approach rather than outcomes that could apply more broadly.

^d^ Busch et al^36^ and Busch et al^37^ are each counted twice in this table (in LAN 3A and 3B) because both studies describe APMs in the 2 categories. However, the total number of study publications in LAN Category 3 (ie, 5), remains unchanged. Beil et al^38^ appears 3 times in this table (in LAN 3A and 3B) because it describes and analyzes 3 different APM implementations.

More than half of the APMs (9 [52.9%]) targeted populations with SUDs, while 4 (23.5%) targeted MH populations, and the rest targeted MH/SUD broadly defined (Table 4). Most APMs focused on adults (11 [64.7%]), while fewer (2 [11.8%]) targeted children or adolescents. Medicaid and publicly funded SUD programs were most common, with each representing 7 APMs (41.2%). Most APM evaluations had an OCEBM rating of 3A (10 [58.8%]), although 2 were prospective controlled trials (1 [5.9%], OCEBM rating of 1; 1 [5.9%], OCEBM rating of 2). The APMs were primarily evaluated on their associations with process-of-care measures (15 [88.2%]), followed by utilization (11 [64.7%]), spending (9 [52.9%]), and clinical outcomes (5 [29.4%]). Only 8 APMs (47.1%) were evaluated for gaming or selection bias. When assessed, statistically significant process-of-care improvements were noted in 12 of 15 APMs (80.0%), while this was only true for 3 of 5 APMs (60.0%) with clinical outcomes and approximately half of the APMs with utilization and spending outcomes (5 of 11 [45.5%] and 4 of 8 [50.0%]). Detailed characteristics and outcomes of the 27 articles and 17 APM implementations are described in Table 5 and the eTable in the Supplement.

**Table 4.** General Characteristics of the Study Publications and APMs Included in this Literature Review

Characteristic	No. (%)	
	Publication (N = 27)	APM (N = 17)
LAN payment category^a^		
2, Fee-for-service with link to quality and value		
A, Foundational payments for infrastructure and operations	1 (3.7)	1 (5.9)
C, Pay-for-performance	9 (33.3)	5 (29.4)
Total	10 (37.0)	6 (35.3)
3, APMs built on fee-for-service infrastructure		
A, APMs with shared savings^b^	4 (14.8)	3 (17.6)
B, APMs with shared savings and downside risk^b^	3 (11.1)	2 (11.8)
Total	7 (25.9)	5 (29.4)
4, Population-based payment		
A, Condition-specific population-based payment	10 (37.0)	4 (23.5)
B, Comprehensive population-based payment	4 (14.8)	2 (11.8)
Total	14 (51.9)	6 (35.3)
Study population insurer type or funding source^c^		
Medicare	3 (11.1)	3 (17.6)
Medicaid	6 (22.2)	7 (41.2)
Patients with dual eligibility	1 (3.7)	1 (5.9)
Publicly funded SUD programs or patients without insurance	15 (55.6)	7 (41.2)
Commercial	3 (11.1)	1 (5.9)
Study population age range		
Child and adolescent	5 (18.5)	2 (11.8)
Adult	20 (74.1)	11 (64.7)
All ages	2 (7.4)	4 (23.5)
Focus outcome category^c^		
MH	5 (18.5)	4 (23.5)
SUD	19 (70.4)	9 (52.9)
MH/SUD	3 (11.1)	5 (29.4)
Specific outcome category assessed^c^		
Processes of care	20 (74.1)	15 (88.2)
Clinical outcomes	5 (18.5)	5 (29.4)
Spending	7 (25.9)	8 (47.1)
Utilization	10 (37.0)	11 (64.7)
Patient dumping, gaming, or adverse selection	11 (40.7)	8 (47.1)
Quality rating^c^^,^^d^		
1, Properly powered and conducted randomized clinical trial; systematic review with meta-analysis	4 (14.8)	1 (5.9)
2, Well-designed controlled trial without randomization; prospective comparative cohort trial	1 (3.7)	1 (5.9)
3, Case-control studies; retrospective cohort study		
3A, Comparison or control group or period, regression analysis, sensitivity analysis	13 (48.1)	10 (58.8)
3B, Comparison or control group or period, regression analysis, no sensitivity analysis	5 (18.5)	4 (23.5)
3C, Comparison or control group or period, no regression or sensitivity analysis	4 (14.8)	4 (23.5)

Abbreviations: APM, alternative payment model; LAN, Learning and Action Network; MH, mental health; SUD, substance use disorder.

^a^ Only LAN categories with examples appear in this table. All LAN categories appear in Table 1.

^b^ Busch et al^36^ and Busch et al^37^ are each counted twice (in LAN 3A and 3B) because both study publications describe APMs in the 2 categories. Beil et al^38^ is counted 3 times (in LAN 3A and 3B) because it describes and analyzes 3 different APM implementations.

^c^ Each study publication (and consequently APM implementation) may include more than 1 study population, insurer type, funding source, study design, focus outcome, or quality rating, thereby contributing to more than 1 category. Consequently, counts for publications and APMs add up to more than 27 or 17, respectively, which remain the denominators for these columns. For the same reasons, percentages may add up to more than 100.

^d^ Adapted from the Oxford Centre for Evidence-Based Medicine Levels of Evidence by subdividing category 3 into 3 levels to account for substantial differences in research design and analysis quality between the studies in this category. Only levels with examples were included; all levels appear in Table 2.

**Table 5.** Summary of 17 APM Implementation Findings Based on Outcomes Examined

APM implementation	LAN category	Outcome category	Source	
			Statistically significant evidence for outcome or significant difference for at least 1 measured outcome	Nonstatistically significant difference, minimal significant difference, or no statistical test provided
Sustaining Healthcare Across Integrated Primary Care Efforts Program	2A	Processes of care	Ross et al,^39^ 2019	NA
		Spending	NA	Ross et al,^39^ 2019
Adolescent Community Reinforcement Approach	2C	Processes of care	Garner et al,^40^ 2011; Garner et al,^41^ 2012; Lee et al,^43^ 2012; Garner et al,^42^ 2018	NA
		Clinical outcomes	Garner et al,^42^ 2018	Garner et al,^41^ 2012
		Spending	Garner et al,^42^ 2018	NA
Spectrum Addiction Services	2C	Processes of care	Shepard et al,^44^ 2006^a^	NA
Outpatient psychosocial counseling treatment center in Maryland	2C	Processes of care	Vandrey et al,^45^ 2011^a^	NA
		Utilization	Vandrey et al,^45^ 2011^a^	NA
		Patient dumping, gaming, or adverse selection	NA	Vandrey et al,^45^ 2011^a^
Washington State Mental Health Integration Program	2C	Processes of care	Bao et al,^47^ 2017; Unutzer et al,^46^ 2012	NA
		Clinical outcomes	Unützer et al,^46^ 2012	NA
		Patient dumping, gaming, or adverse selection	Unützer et al,^46^ 2012	NA
Connecticut’s Behavioral Health Partnership	2C	Processes of care	Schmutte et al,^48^ 2019^a^	NA
Medicare Shared Savings Program Accountable Care Organizations^b^	3A	Processes of care	Busch et al,^36^ 2016; Busch et al,^37^ 2017	NA
		Clinical outcomes	NA	Busch et al,^36^ 2016
		Spending	NA	Busch et al,^36^ 2016
		Utilization	NA	Busch et al,^36^ 2016
		Patient dumping, gaming, or adverse selection	Busch et al,^36^ 2016	NA
Maine Medicaid Accountable Communities Initiative^c^	3A	Processes of care	NA	Beil et al,^38^ 2019
		Spending	NA	Beil et al,^38^ 2019
		Utilization	NA	Beil et al,^38^ 2019
Vermont Medicaid Shared Savings Program^c^	3A	Processes of care	NA	Beil et al,^38^ 2019
		Spending	Beil et al,^38^ 2019	NA
		Utilization	Beil et al,^38^ 2019	NA
Medicare Pioneer Accountable Care Organizations^b^	3B	Processes of care	Busch et al,^37^ 2017	Busch et al,^36^ 2016
		Clinical outcomes	NA	Busch et al,^36^ 2016
		Spending	Busch et al,^36^ 2016	NA
		Utilization	NA	Busch et al,^36^ 2016
		Patient dumping, gaming, or adverse selection	Busch et al,^36^ 2016	NA
Minnesota Integrated Health Partnerships Program^c^	3B	Processes of care	Beil et al,^38^ 2019	NA
		Spending	NA	Beil et al,^38^ 2019
		Utilization	Beil et al,^38^ 2019	NA
Delaware Division of Substance Abuse and Mental Health–Outpatient Services APM	4A	Processes of care	Stewart et al,^49^ 2013	McLellan et al,^50^ 2008^a^
		Utilization	NA	McLellan et al,^50^ 2008^a^
		Patient dumping, gaming, of adverse selection	NA	McLellan et al,^50^ 2008^a^; Stewart et al,^49^ 2013
Delaware Division of Substance Abuse and Mental Health–Detoxification Care Transition APM	4A	Utilization	NA	Haley et al,^51^ 2011^a^
		Patient dumping, gaming, or adverse selection	Haley et al,^51^ 2011^a^	NA
Maine Addiction Treatment System, phase 1 of performance-based contracting	4A	Clinical outcomes	Commons et al,^52^ 1997^a^	NA
		Utilization	Commons et al,^52^ 1997^a^	NA
		Patient dumping, gaming, or adverse selection	Shen,^56^ 2003; Lu et al^54^ 2003; Lu,^53^ 1999^a^; Lu and Ma,^55^ 2006	NA
Maine Addiction Treatment System, phase 2 of performance-based contracting	4A	Processes of care	NA	Brucker and Stewart,^57^ 2011^a^; Stewart et al,^58^ 2018
		Utilization	NA	Brucker and Stewart,^57^ 2011^a^
		Patient dumping, gaming, or adverse selection	NA	Stewart et al,^58^ 2018
BCBSMA Alternative Quality Contract	4B	Processes of care	Barry et al,^59^ 2015; Stuart et al,^60^ 2017	NA
		Spending	Barry et al,^59^ 2015	Stuart et al,^60^ 2017; Donohue et al,^61^ 2018
		Utilization	Barry et al,^59^ 2015; Stuart et al,^60^ 2017	Donohue et al,^61^ 2018
Oregon Coordinated Care Organizations	4B	Processes of care	NA	Rieckmann et al,^62^ 2018^c^

Abbreviations: APM, alternative payment model; BCBSMA, Blue Cross/Blue Shield of Massachusetts; LAN, Learning and Action Network; NA, not applicable.

^a^ Publication had a quality rating of 3B or 3C according to the Oxford Centre for Evidence-Based Medicine Levels of Evidence modified for this investigation (Table 2).

^b^ Busch et al^36^ and Busch et al^37^ were counted twice (in LAN 3A and 3B) because both study publications describe APMs in the 2 categories.

^c^ Beil et al^38^ was counted 3 times (in LAN 3A and 3B) because it describes and analyzes 3 different APM implementations.

### Association of APMs With Outcomes

#### LAN 2A: Foundational Payments for Infrastructure and Operations

The Sustaining Healthcare Across Integrated Primary Care Efforts (SHAPE) study^39^ (OCEBM rating, 3A) evaluated the association of LAN 2A foundational payments with integrating MH/SUD services in primary care. Patients in the APM group were 3.5 times more likely to be screened for depression, 1.5 times more likely to be diagnosed with depression, and 1.3 times more likely to be diagnosed with anxiety. There were no differences for SUD found. Additionally, results showed a total cost savings of $17 per member per month and a net savings of more than $1 million during the study period.

#### LAN 2C: Pay-for-Performance

A total of 9 articles^40,41,42,43,44,45,46,47,48^ assessed 5 unique APM implementations (LAN 2C), 1 of which was a national, Substance Abuse and Mental Health Service Administration–funded pay-for-performance initiative using a cluster randomized clinical trial design that was evaluated in 4 separate publications,^40,41,42,43^ all with OCEBM ratings of 1. This program aimed to improve the implementation of the Adolescent Community Reinforcement Approach, an evidence-based SUD treatment model. The APM intervention resulted in therapists being more likely to demonstrate competence in delivering evidence-based treatment and in patients being more likely to receive the targeted dose,^41^ consistent with an earlier study examining therapist-level implementation of this treatment.^40^ Patients in the APM were also more likely to initiate SUD treatment, although there was no change in engagement.^43^ Findings were mixed on clinical outcomes, with 1 publication^41^ reporting no change in remission status, while another^42^ noted that receiving the targeted dose of the treatment (which was more likely in the APM group) was associated with a greater percentage of days abstinent. Additionally, patients in the APM group had lower MH/SUD treatment spending but higher spending when accounting for the cost of the APM itself.^42^

Two studies^46,47^ examined an APM for MH/SUD integration that was implemented across a network of community health clinics in Washington state, both with OCEBM ratings of 3A. One study^47^ found that the APM was associated with a higher likelihood of having at least 1 follow-up contact, psychiatric case review, and Patient Health Questionnaire–9 assessment. Another study^46^ demonstrated that the APM was associated with more rapid depression symptom improvement as well as a higher likelihood of achieving treatment response and timely follow-up.

Three other LAN 2C studies^44,45,48^ evaluated separate APMs. In a 2006 study,^44^ a counselor-level APM aiming to improve patient SUD treatment attendance (OCEBM rating, 3B) was associated with higher odds of completing at least 5 sessions but not the target number of 12 sessions. Another APM at a community SUD treatment clinic^45^ (OCEBM rating, 3C) was associated with patients attending more visits in the first 30 days and with higher 90-day treatment program retention without evidence of gaming. A third study examining the association of an APM with pediatric psychiatric hospitalization among children and adolescents with Medicaid coverage in Connecticut^48^ (OCEBM rating, 3C) found a nearly 25% decrease in length of stay in the final APM year compared with baseline, but no change in 7-day or 30-day readmission rates. While these latter 2 studies reported positive results, both were in OCEBM category 3C for study design quality.

#### LAN 3A: APMs With Shared Savings

In 2012, CMS implemented the Medicare Shared Savings Program (SSP) Accountable Care Organization (ACO) program.^63^ The SSP was primarily a 1-sided ACO model (ie, organizations share financial gains but not losses).^64,65^ In a series of 2 studies,^36,37^ both with OCEBM ratings of 3A, no differences in process-of-care outcomes, health care spending, service utilization, or patient characteristics relative to the comparison group (suggesting no selection bias) were found.^36^ Additionally, among beneficiaries diagnosed with depression, the SSP was associated with a decrease of 0.5% in antidepressant use and an increase of 0.4 days in the portion of days covered.^37^

#### LAN 3B: APMs With Shared Savings and Downside Risk

Alongside the SSP in 2012, CMS implemented the Pioneer ACO. Unlike the SSP, it initially included 2-sided risk (ie, organizations share financial gains and losses), and CMS preferentially selected organizations with existing infrastructure for case management and coordination.^66,67^ In a 2016 analysis^36^ (OCEBM rating, 3A), the Pioneer ACO was associated with a slight reduction in MH admissions and total MH spending in the first year of the program but not in the second. However, no changes were noted in utilization or processes-of-care outcomes. Among beneficiaries with depression, the Pioneer ACO was associated with slight increases in days of medication supplied in the first year and slight increases in the proportion of days supplied with antidepressants in the first 2 years. In a related study^37^ (OCEBM rating, 3A), there was no demonstrated change in the proportion of Pioneer ACO beneficiaries with depression using an antidepressant. Additionally, there was some evidence that the Pioneer ACO was associated with a reduction in the percentage of beneficiaries with MH diagnoses, although it is unlikely that these shifts contributed to reduced MH admissions and is unclear whether this finding represented intentional efforts.^36^

Another study examined the associations of 3 different Medicaid ACOs with outcomes in MH/SUD populations in Maine (LAN 3A), Minnesota (LAN 3B), and Vermont (LAN 3A)^38,68,69,70^ (OCEBM rating, 3A). Minnesota’s ACO was associated with an increase in all-cause inpatient admissions in the MH/SUD population; Vermont’s ACO was associated with a decrease; and Maine’s ACO was associated with no change. All 3 ACOs were associated with reductions in emergency department visits, although none with changes in 30-day readmission rates. Maine’s ACO uniquely demonstrated a process-of-care measure improvement, finding an increase in 180-day antidepressant adherence among beneficiaries with depression. Vermont’s ACO was associated with lower total spending among beneficiaries with MH disorders, making it the only Medicaid ACO to hold that distinction.

#### LAN 4A: Condition-Specific Population-Based Payment

Delaware implemented a LAN 4A APM in 2002, providing financial incentives to publicly funded outpatient SUD programs; 1 outcome measure was aimed at improving capacity utilization (ie, proportion of treatment capacity used), while 2 measures targeted active participation and completion of treatment.^50^ This APM was associated with reduced wait times and increased length of stay in the treatment programs (OCEBM rating, 3A).^49^ It was also associated with greater average SUD treatment capacity utilization and a higher proportion of patients meeting active participation requirements during a period when the population appeared to become more severely ill (OCEBM rating, 3B).^50^ In 2008, Delaware implemented a new LAN 4A APM, incentivizing the state’s sole detoxification vendor to maintain current detoxification unit capacity (of ≥90%) and increase the proportion of frequent detoxification patients who transition to SUD care after detoxification (OCEBM rating, 3C).^51^ While detoxification capacity was maintained and the rates of transition to outpatient care improved, only 8% of postdetoxification rehabilitation treatment episodes met the targeted length of stay (60 outpatient days or 30 residential days for most patients). There was no evidence of adverse selection.^51^

Another group of 7 studies^52,53,54,55,56,57,58^ examined 2 iterations of a LAN 4A APM for SUD treatment in Maine. In the first phase, organization-level performance data were reviewed annually, leading to possible positive or negative contractual changes in the subsequent year. This version of the APM was associated with improvements in the efficiency and effectiveness of SUD programs (OCEBM rating, 3B).^52^ However, 2 subsequent evaluations (OCEBM ratings, 3B^53^ and 3A^55^) found that the APM was also associated with increases in gaming. Additionally, there was some evidence for selection bias, with the APM being associated with a lower proportion of clients with higher severity illness in ambulatory settings (OCEBM rating, 3A).^56^ Results of another evaluation^54^ found that the APM was associated with better clinician-patient matching and higher rates of referral (OCEBM rating, 3A), contrasting the earlier findings of possible patient dumping via selection bias. Building off these experiences, Maine subsequently implemented a second-generation APM that included base payments, incentive payments (including penalties), and quarterly (as opposed to annual) assessments. Two separate analyses (OCEBM ratings, 3B^57^ and 3A^58^) found that the revised APM was not associated with any changes in process-of-care measures or utilization; there was also no evidence for selection bias.

#### LAN 4B: Comprehensive Population-Based Payment

Three study publications^59,60,61^ (all OCEBM rating 3A) assessed the 3-year MH/SUD outcome associations of the Blue Cross/Blue Shield of Massachusetts Alternative Quality Contract (AQC), a LAN 4B commercial insurance ACO. The AQC compensated participating organizations via a risk-adjusted, prospective payment for all primary and specialty care, with bonuses available based on performance across 64 measures (most unrelated to MH/SUD care). Only 5 of the initial 12 AQC contracts included risk for MH/SUD services. The AQC was associated with slightly less MH service use among organizations with downside risk for MH/SUD but not among those without.^59^ The AQC was associated with no change in MH/SUD spending in organizations with and without downside MH/SUD risk.^59^ Among organizations facing MH/SUD risk, the AQC was associated with no change in SUD service use, spending, or quality measures.^60^ In organizations without MH/SUD risk, the AQC was associated with no change in engagement, a slight decrease in SUD medication use, and slight increases in the probability of SUD service use and treatment initiation. Overall, including organizations with and without MH/SUD risk, the AQC was not associated with any changes in SUD medication use or spending.^61^

Implemented in 2012, Oregon’s Medicaid Coordinated Care Organizations were a LAN 4B APM implementation that included a prospectively paid global budget for physical health, MH/SUDs, and dental care. All participating organizations had the opportunity to receive annual incentive payments from a quality pool based on performance across 17 quality metrics (not all related to MH/SUD). The APMs have been associated with improved rates of screening and brief intervention but no changes in SUD identification or treatment initiation (OCEBM rating, 3B)^62^; additionally, screening has been associated with changes in initiating SUD treatment 6 or 12 months after implementation.^62^

## Discussion

We identified 17 distinct APMs that were evaluated for their associations with MH/SUD care delivered across diverse settings and patient populations. Included APMs had a range of funding sources (eg, Medicare, Medicaid, state public health systems, commercial insurance) and covered various diagnostic groups (eg, severe mental illness, depression, SUDs) and age categories (child to adult). No single LAN category predominated the literature. More than 70% of APMs (12 of 17) underwent methodologically rigorous evaluations (ie, had OCEBM ratings of 3A or higher).

Process-of-care measures predominated in the evaluations (nearly 90%), with direct clinical outcomes being examined much less commonly (nearly 30%). In an important note of caution, gaming of outcomes was noted in 1 APM,^53,54,55^ and 3 APMs^49,50,51^ were associated with changes in patient composition that could suggest selection bias, possibly erroneously inflating findings of improvement in some cases (although these changes were likely inconsequential in others^36^). Overall, only 8 of 17 APMs (47.1%) were evaluated for gaming or selection bias in any capacity.

Among APMs demonstrating statistically significant results, some were large and clinically meaningful, while others were smaller, of more questionable clinical significance, or mixed in terms of clinical benefit. For example, the SHAPE APM, which integrated MH/SUD services into primary care, was associated with 3.5 times higher rates of screening for depression as well as clinically significant increases in the identification of depression and anxiety (1.5 times and 1.3 times higher, respectively).^39^ In contrast, while there were some positive results associated with the Massachusetts AQC among organizations in the APM who were not at financial risk for MH/SUD care (ie, higher probability of any SUD service use and increased SUD identification), the magnitudes of these increases were small, and they were coupled with some less desirable process-of-care outcome findings (ie, decreased SUD medication use and SUD care initiation).^60^

Additionally, associations that seem small in nature may actually be clinically significant based on the prevalence of a condition or scale of the APM. For example, in the 2013 Medicare Shared Savings Program, the APM was associated with a decrease of 0.5% in antidepressant use and, among those taking antidepressants, an increase of 0.4 days in the proportion of days covered, both of which were statistically significant.^37^ Although the magnitudes appeared relatively small, the prevalence of depression, coupled with the fact that this was a large-scale evaluation of a Medicare APM, suggests that this intervention could have had a substantial population effect.

Our findings highlight important gaps in the literature on APMs in MH/SUD care. First, we found few studies examining the effects of APMs on the MH/SUD care of children or adolescents. Second, although process-of-care measures are expected to be associated with clinical outcomes and are commonly used in APM evaluations for their feasibility, the association between these 2 outcome types is not always certain, often leaving the associations between APMs and clinical outcomes unclear. Although challenging,^71,72^ future work is needed to identify target clinical outcomes for APMs that are both clinically useful and pragmatic, particularly for those evaluated across systems. Third, given the complexity and varied characteristics of APMs, further research is needed to learn the key components of successful APMs through mixed methods. Fourth, fewer than half the APMs were evaluated for gaming or adverse selection; such evaluations should be routine to ensure that an apparent positive association of an APM is not a false-positive that was achieved through selection effects in the study population. To better inform policy and practice, future APM investigations should have at least the following elements: (1) rigorous design and analysis (ie, either a randomized clinical trial or natural experiment that includes robust observational design methods and sensitivity analyses, as in OCEBM category 3A), (2) an evaluation for possible gaming or adverse selection, (3) at least 1 clinical outcome, and (4) process-of-care measures that incentivize fidelity to evidence-based care (eg, collaborative care for depression^46^).

It is noteworthy that qualitative themes and barriers identified in this systematic review were overwhelmingly from evaluations of ACOs (LAN 3 and 4) that spanned a variety of payers and populations, including Medicare, Medicaid, and commercial insurance.^59,60,61,73,74^ Some of these findings are likely generalizable beyond ACOs. For example, in earlier ACOs that were not designed solely to manage MH/SUD care, these services were not considered low-hanging fruit and were often not an early focus of coordination or integration efforts.^73,74^ It is therefore unsurprising that changes in MH/SUD process-of-care, utilization, or spending outcomes were limited or mixed in the early ACOs. We would expect similar findings in APMs other than ACOs that were not primarily intended to manage MH/SUD care.

Additionally, qualitative findings from articles in this review demonstrate that even advanced APMs managed by well-resourced health systems may experience challenges in lowering utilization or spending, have difficulty improving care quality consistently over time, and require infrastructure investments. Although qualitative findings from ACO-focused evaluations offered some broadly applicable insights, the lack of comparable findings from evaluations of other APM categories is a notable gap in the literature.

### Limitations

This study has limitations. Our findings are limited by the restriction to empirical evaluations with comparison or control groups, which may have missed descriptive or perspective publications on APMs. This decision was made to limit our review to the most methodologically rigorous studies. However, despite our exclusion criteria, other prior reviews have been less comprehensive.^15,18^

## Conclusions

While APMs were often associated with improvements in process-of-care measures, they were less often associated with reduced MH/SUD utilization and spending. Additionally, more information is needed on clinical outcomes, which have rarely been evaluated in APMs. Studies to date have found some evidence for gaming and adverse selection in the APM context, highlighting the importance of including assessments for these potential phenomena in all future evaluations. Further research is needed to better understand the varied qualitative experiences across APMs, their successful ingredients, and the associations of these models with clinical outcomes across diverse populations and settings.

## Notes

Supplement. **eAppendix.** Detailed Description of Literature Review **eFigure.** Study Flow Diagram **eTable. **Outcomes From 27 Study Publications Assessing 17 LAN-Defined APMs **eReferences.**
