# Supplementary material for: Association of Alternative Payment and Delivery Models With Outcomes for Mental Health and Substance Use Disorders: A Systematic Review
Source: JAMA Netw Open. 2020 Jul 23;3(7):e207401. doi: 10.1001/jamanetworkopen.2020.7401 (PMC7378751; doi:10.1001/jamanetworkopen.2020.7401)
Supplement: Supplement. — eAppendix. Detailed Description of Literature Review eFigure. Study Flow Diagram eTable. Outcomes From 27 Study Publications Assessing 17 LAN-Defined APMs eReferences. [file jamanetwopen-3-e207401-s001.pdf]

Supplementary Online Content

Carlo AD, Benson NM, Chu F, Busch AB. Association of alternative payment and delivery models with outcomes for mental health and substance use disorders: a systematic review. *JAMA Netw Open*. 2020;3(7):e207401. doi:10.1001/jamanetworkopen.2020.7401

eAppendix. Detailed Description of Literature Review
eFigure. Study Flow Diagram
eTable. Outcomes From 27 Study Publications Assessing 17 LAN-Defined APMs
eReferences.

This supplementary material has been provided by the authors to give readers additional information about their work.

© 2020 Carlo AD et al. *JAMA Network Open.*

eAppendix. Detailed Description of Literature Reviews:

Pilot Literature Review:
Prior to the final literature search, the authors conducted a pilot search and systematic review. This occurred on September 1, 2017 and involved searches of MEDLINE (via Pubmed), Scopus, and Business Source Complete for articles dating from January 1, 1997. Search terms included "accountable care," "bundled payment," "alternative payment," and "pay-for-performance," along with "mental health" and "behavioral health." The following MeSH terms were also searched: "Accountable Care Organizations," "Patient Care Bundles," "Insurance, Health, Reimbursement," "Value-Based Purchasing," "Value-Based Insurance," "Behavioral Medicine," "Psychiatry," or "Mental Health Services." This pilot search yielded 555 articles, 472 of which remained after de-duplication. Two reviewers (ADC, NMB) independently assessed half of the articles, while systematically examining all articles excluded by the other reviewer. All discrepancies were resolved through email discussions and meetings. A third, senior reviewer (ABB), participated in meetings and reviewed articles independently upon request to confirm inclusion/exclusion in the systematic review. Ultimately, 435 articles were excluded and 37 met all of the inclusion criteria. However, upon review of citations in the examined articles and discussions with content experts, the authors found that numerous potentially relevant articles had not appeared in the initial search, particularly those related to performance-contracting and substance use disorders. It was also determined that some of the articles initially thought to meet inclusion criteria were not alternative payment models (APMs) according to the Health Care Payment Learning and Action Network's (LAN) APM framework[1,2].

Final Literature Review:
Consequently, the authors iteratively re-examined search terms, added an additional database (PsychInfo) and reconducted the search on April 15, 2018. This final literature search included the MEDLINE (via PubMed), PsychInfo, Scopus and Business Source databases and was restricted to studies between January 1, 1997 and April 15, 2018. Search terms included all of those stated above, with the additions of "performance contracting" and "substance use disorders." Additionally, the authors (ADC, NMB) manually searched reference lists of pertinent articles identified through the systematic review for relevant citations that were potentially missed. The primary focus of the literature review was articles on comparative studies examining the impact of alternative payment models on mental health clinical and process-of-care outcomes. For inclusion, articles had to describe studies from the United States, be written in English, examine an alternative payment model for mental health or substance use disorder services (defined according to the Health Care Payment Learning and Action Network's (LAN) APM framework[1,2]), assess a defined mental health or substance use disorder outcome, and have a comparison or control group or period. The authors included randomized-controlled trials (RCT), non-randomized controlled studies (NRS) (e.g., quasi-experimental studies and natural experiments), and pre-post studies. This literature review identified 1,015 articles, though only 924 remained after removal of duplicates). Ultimately, 20 articles met all inclusion criteria. Of note, 13 articles were excluded because they evaluated LAN 4N APMs (i.e., payments were not tied to quality or value).

Literature Review Update:
On May 17, 2019, the literature review was updated, as it had been more than one year since the previous search. This updated literature search included the MEDLINE (via PubMed), PsychInfo, Scopus, and Business Source databases and was restricted to studies between April 15, 2018 and May 17, 2019. The search terms, inclusion criteria, and exclusion criteria were identical to the final literature review described above. This search initially yielded 75 articles, though two were noted to also be in the previous search, leaving a total of 73 new articles. Even though the time periods did not overlap, articles appeared in both searches because PsychInfo, Scopus, and Business Source did not allow search queries to be specified to intervals smaller than one year, allowing articles published between January 1, 2018 and April 15, 2018 to appear twice. This led to a combined total of 1,088 articles from the final and updated searches, though only 986 articles remained after removal of duplicates. Of the new 73 articles appearing in the literature review update, 11 were found to be duplicates. Ultimately, 62 new articles were identified, with 7 meeting all inclusion criteria. Of note, no articles in this search were found to have evaluated LAN 4N APMs. This led to a combined total of 27 included articles from the final and updated searches. See Figure S1 below for the Preferred Reporting Items for Systematic Reviews and Meta-Analyses (PRISMA) diagram.

Three authors (ADC, NMB, ABB) reviewed all of the alternative payment model (APM) publications and determined the Oxford Centre for Evidence-based Medicine (OCEBM) and Learning and Action Network (LAN)

© 2020 Carlo AD et al. *JAMA Network Open.*

categorizations. Discrepancies were resolved by email and meeting discussions until 100% agreement was achieved among the three reviewers.

© 2020 Carlo AD et al. *JAMA Network Open.*

eFigure. Study Flow Diagram

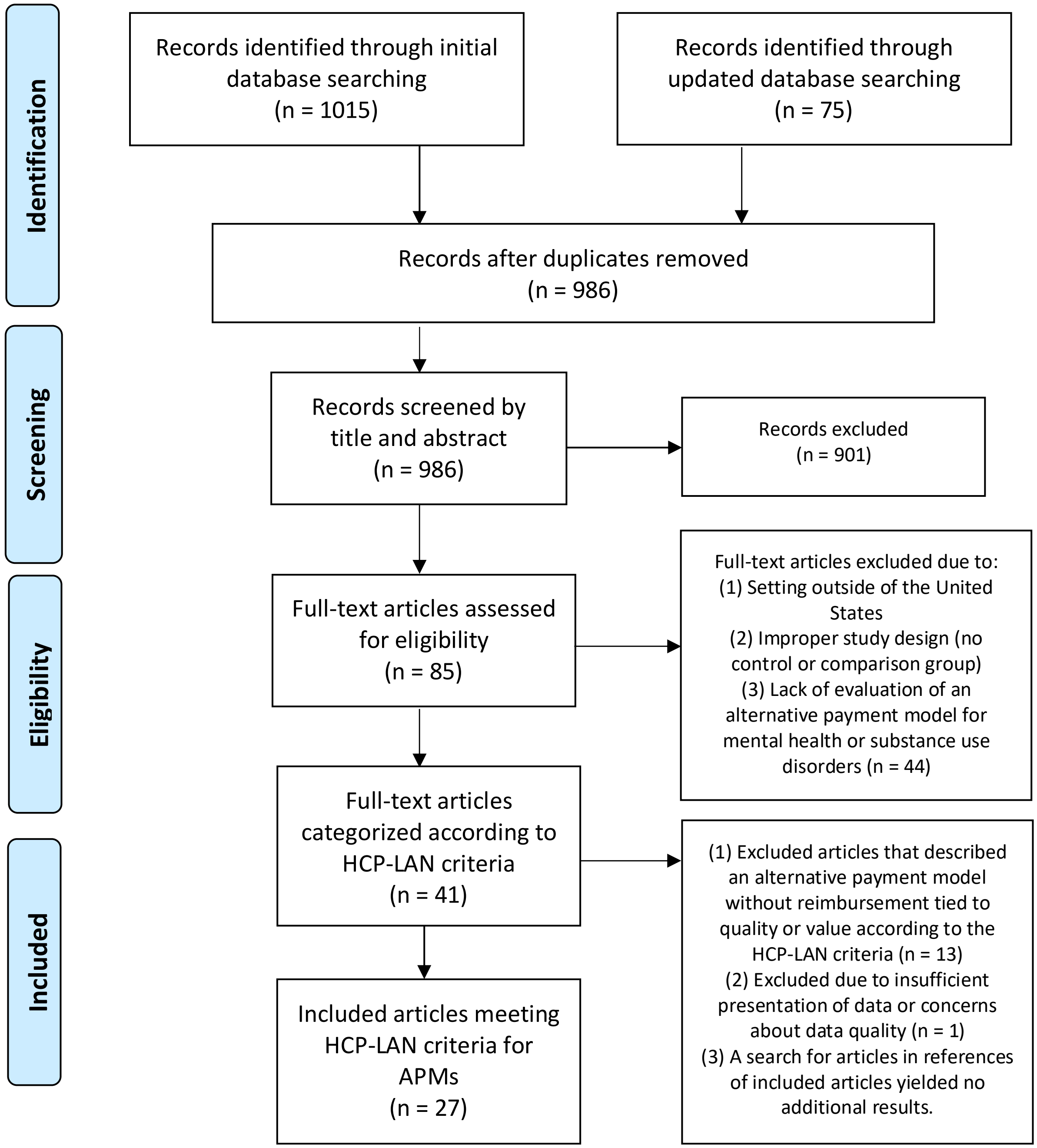

© 2020 Carlo AD et al. *JAMA Network Open.*

eTable. Outcomes From 27 Study Publications Assessing 17 LAN-Defined APMs

APM	Payment Model	Intervention	Comparison Group	Study Publication	N	Outcome	Findings and Direction of Effect
Sustaining Healthcare Across Integrated Primary Care Efforts (SHAPE) Program	LAN 2A	Lump sum payments for program implementation - Colorado's Rocky Mountain Health Plans provided flexible, non-fee-for-service funding to facilitate behavioral health integration; practice-level payment amounts were calculated using an activity-based costing method designed to account for anticipated program costs	Practices without foundational payments	Ross et al, 2018[3]	Intervention: 3 practices (81,900 annual patient visits); Comparison: 3 practices (81,536 annual patient visits)	Processes-of-Care	Relative to the comparison group, the APM was associated with increased rates of depression screening (RR 3.5, 95%CI 3.0 to 4.2, p < 0.0001), depression diagnosis (RR 1.5, 95%CI 1.3 to 1.8, p < 0.0001), and anxiety diagnosis (RR 1.3, 95%CI 1.1 to 1.6, p = 0.001). There was no significant change in the rate of SUD diagnosis.
						Spending	The APM was associated with an estimated net savings of $1.08 million over the study period.
Adolescent Community Reinforcement Approach (A-CRA)	LAN 2C	Pay-for-performance financial incentives for therapists within SUD treatment organizations - in addition to their normal compensation, therapists could earn: (A) $50 for each month a randomly selected session audio recording was rated above minimum A-CRA competency or (B) $200 for each adolescent to whom they delivered the targeted threshold level of A-CRA treatment. Payments were made on a monthly basis	Therapists or organizations without pay-for-performance	Garner et al, 2011[4]	Intervention: 14 organizations (47 therapists); Comparison: 15 organizations (48 therapists)	Processes-of-Care	The APM was associated with increases in therapists' intentions to achieve monthly competence (β=1.19, SE=0.32, p=0.001) and deliver a targeted threshold level of treatment to clients (β=1.11, SE=0.31, p=0.002), both as measured on a 7-point Likert-type scale.
				Garner et al, 2012[5]	Intervention: 14 organizations, 60 therapists and 539 patients; Comparison: 15 organizations, 60 therapists and 634 patients	Processes-of-Care	APM therapists had a higher likelihood of demonstrating A-CRA competence (RR 2.24, 95%CI 1.12 to 4.48, P=0.02) and patients in the intervention group had higher odds of receiving target A-CRA (OR 5.19, 95%CI 1.53 to 17.62, P=0.01).
						Clinical Outcomes	The APM was not associated with any significant difference in patient-level remission.
				Garner et al, 2018[6]	Intervention: 14 organizations, 60 therapists and 539 patients; Comparison: 15 organizations, 60	Processes-of-Care	Relative to the comparison, APM organizations had a higher average number of months that therapists demonstrated A-CRA competence (mean (SD)=8.62 (7.58) vs. 18.64 (14.68), p < 0.001, 116% increase) and a higher average number of patients

© 2020 Carlo AD et al. *JAMA Network Open.*

APM	Payment Model	Intervention	Comparison Group	Study Publication	N	Outcome	Findings and Direction of Effect
					therapists and 634 patients		who received target A-CRA (2.27 (2.74) vs. 9.64 (1.31), p < 0.001, 325% increase).
						Clinical Outcomes	Patients who received target A-CRA reported a significantly greater percentage of days of abstinence from alcohol and drug use (β=0.153, SE=0.076, p < .05).
						Spending	Relative to the comparison, APM organizations had higher total spending (mean (SD) = $62,917 ($22,953) vs. $66,256 ($25,006), p<0.001), though treatment spending was lower ($44,073 ($22,951) vs. $39,838 ($15,051), p<0.001). Relative to the comparison, the incremental cost-effectiveness ratio for APM therapist months of A-CRA competence, patients receiving target A-CRA, and days of abstinence per patient for the intervention group were $333, $453, and $8.134, respectively; the incremental cost per quality adjusted life year (QALY) for patients was $8,681.
				Lee et al, 2012[7]	Intervention: 14 Organizations, 60 therapists and 539 patients: Comparison: 15 Organizations, 60 therapists and 634 patients	Processes-of-Care	APM intervention site adolescents entering the program after implementation of the APM were significantly more likely to initiate treatment (β=0.311, SE=0.140, 95%CI 0.036 to 0.586, p<0.05), but not effectively engage in treatment, both as determined from initiation interaction terms (APM site × Client admitted post-APM implementation).
Spectrum Addiction Services	LAN 2C	Pay-for-performance financial incentives for therapists - therapists could earn, in addition to their normal compensation, bonuses of: (A) $100 for each client who attended 5 sessions, and (B) $50 for	Pre-intervention phase	Shepard et al, 2006[8]	11 counselors followed before and after implementation of financial incentives; 123 total clients were treated	Processes-of-Care	Relative to the comparison, clients in the APM period had higher odds of completing at least 5 sessions of treatment (OR 4.12, β=1.4166, p=0.0014), but not 12 sessions of treatment.

© 2020 Carlo AD et al. *JAMA Network Open.*

APM	Payment Model	Intervention	Comparison Group	Study Publication	N	Outcome	Findings and Direction of Effect
		each client who attended the full course of 12 sessions					
Outpatient Psychosocial Counseling Treatment Center in Maryland	LAN 2C	Pay-for-performance financial incentives for therapists - therapists could earn, in addition to their normal compensation, bonuses for: (A) attendance ($10 for each client who attended 5-6 therapy sessions in 1 month and $25 for each client who attended 7+ sessions) and (B) retention (quarterly cash payments of $100, $150, $175 or $200 if the retention rate of each 3-month cohort of their clients met benchmarks of 65%, 75%, 85%, or 95% retention, respectively)	Pre-intervention phase	Vandrey et al, 2011[9]	7 of 11 counselors (with more than 2 clients) participated during the baseline phase, and 10 of 11 during the intervention phase; 426 total clients were treated (165 in baseline and 261 in intervention)	Processes-of-Care	Relative to the comparison, overall 90-day retention improved in the APM period (40% vs. 53%, χ^2=4.846, p < 0.05), with improvements noted for all but one of the counselors in the study. Further, improved 90-day retention was mediated by early treatment attendance rates of individual patients (Sobel = 3.18, p<0.05).
						Utilization	Relative to the pre-intervention period, the average number of treatment sessions attended during the first 30 days increased during the APM phase (mean (SD) = 4.6 (2.7) vs. 5.5 (2.9), t=−3.40, p<0.01). An increase in sessions attended was observed for all counselors (with the exception of one) with at least 5 clients.
						Patient Dumping/ Gaming/ Adverse Selection	There were no pre- and post-intervention patient population differences in gender, race/ethnicity, treatment referral source (from criminal justice system), or primary drug of concern entering treatment.
Washington State Mental Health Integration Program (MHIP)	LAN 2C	Pay-for-performance for collaborative care management - Prior to 2009, the Community Health Plan of Washington (CHPW) fully reimbursed participating primary care clinics for the costs associated with care coordinators for CoCM. When the APM went into effect on January 1, 2009, 25% of annual CoCM program	Patients not exposed to value-based payment	Unützer et al, 2012[10]	Intervention: 1,673 depressed adults enrolled in collaborative care and exposed to value-based payment; Comparison: 6,304 depressed adults enrolled in collaborative care that were not exposed to value-based payment	Processes-of-Care	For patients non-exposed vs. exposed to the APM: follow-up contacts within 2 weeks after initial assessment (42.4% vs. 59.3%, p<0.001), follow-up contacts within 4 weeks after initial assessment (52.6% vs. 71.8%, p<0.001), number of follow-up contacts in first 4 weeks after initial assessment (mean (SD) = 0.97 (1.19) vs. 1.42 (1.30), p<0.001), total number of follow-up contacts during treatment (6.17 (8.63) vs. 5.54 (6.76), p=0.002) and any psychiatric consultation during treatment (49.4% vs. 59.8%, p<0.001)

© 2020 Carlo AD et al. *JAMA Network Open.*

APM	Payment Model	Intervention	Comparison Group	Study Publication	N	Outcome	Findings and Direction of Effect
		funding became contingent on meeting quality indicators, such as timely follow-up, psychiatric consultation for patients not showing clinical improvement, and regular tracking of psychotropic medications				Clinical Outcomes	Relative to the non-exposed, the APM-exposed group had higher likelihood of achieving treatment response (HR 1.73, 95%CI 1.39 to 2.14, p<0.001). Additionally, between the pre- and post-APM periods, the median time elapsed for reaching depression treatment response was reduced from 64 to 25 weeks.
						Patient Dumping/ Gaming/ Adverse Selection	Relative to the non-exposed group, the APM-exposed group was slightly younger (mean (SD) = 41.9 years old (11.0) vs. 41.1 (12.2), p=0.014) and more female (48% vs. 52%, p=0.004). Additionally, relative to the non-exposed group, the APM-exposed group had higher percentages of patients with comorbid anxiety (51% vs. 67%, p<0.001), PTSD (16% vs. 23%, p<0.001), and cognitive disorder (1.4% vs. 2.4%, p=0.012).
				Bao et al, 2017[11]	Intervention: 1,250 depressed adults enrolled in collaborative care who were exposed to value-based payment for at least 1 month; Comparison: 556 depressed adults enrolled in collaborative care who were never exposed to value-based payments	Processes-of-Care	The APM was associated with increased probabilities of follow-up contact (9% greater, β=0.05, 95%CI 0.00 to 0.10, p<0.05), psychiatric consultation (30% greater, β=0.04, 95%CI 0.00 to 0.07, p<0.05) and PHQ-9 assessment (15% greater, β=0.07, 95%CI 0.02 to 0.11, p<0.05).
Connecticut's Behavioral Health Partnership	LAN 2C	Financial incentives for Connecticut hospitals to reduce pediatric psychiatry length of stay - participating hospitals were awarded a share of	Pre-intervention phase	Schmutte et al, 2019[12]	Medicaid-covered youths at eight hospitals in Connecticut in 2008 (715 patients), 2009	Processes-of-Care	Relative to the 2007 baseline period, the average inpatient length of stay decreased by 24.9% (18.1 vs. 13.6 days, z=7.04, p<0.001) by the end of the APM intervention in 2010. Readmission rates at 7- and 30-days

© 2020 Carlo AD et al. *JAMA Network Open.*

APM	Payment Model	Intervention	Comparison Group	Study Publication	N	Outcome	Findings and Direction of Effect
		a performance fund if they were able to demonstrate achievement of case-mix adjusted goals for length of stay reduction (or maintenance of already efficient length of stay) and enhanced family engagement in care			(1,408 patients), and 2010 (782 patients)		after discharge were not significantly different between the baseline and APM intervention periods.
Medicare Shared Savings Program (SSP) Accountable Care Organizations (ACOs)	LAN 3A	ACO beneficiaries - SSP Track 1 ACO organizations (one-sided risk - organizations share in savings, but not losses) were eligible to receive shared savings on first dollar (i.e., applies to an ACO's total savings below its benchmark) once a certain savings threshold, the minimum savings rate was achieved. The minimum savings rate varied across organizations based on beneficiary count and ranged from 2.0% to 3.9%[13,14]. Of note, sharing savings details differed across the initial years of the SSP program implementation.	Non-ACO beneficiaries	Busch et al, 2016[15]	All person-year sample size estimates reported are in the pre-intervention period and are for beneficiaries with mental illness.<br><br>2012 entrant SSP ACO group: 79,993 person-years, control group: 864,672 person-years.<br><br>2013 entrant SSP ACO group: 84,099 person-years, comparison group: 1,189,577 person-years.	Processes-of-Care	For the 2013 SSP entrants, none of the process-of-care outcomes (30-day mental health readmissions, outpatient mental health follow-up within 7 days of discharge, or identified as having a depressive disorder) were statistically significant. For the 2012 SSP entrants, there was a slight decrease in identifying a depressive disorder from the pre-contract annual mean of 4.7% (β=-0.3 percentage points, p<0.05), but no significant decrease in 30-day mental health readmissions from the pre-contract annual mean.
						Clinical Outcomes	No evidence that the SSP impacted mental health status based on Medicare beneficiary self-report (measured by the Consumer Assessment of Healthcare Providers and Systems)
						Spending	There were no statistically significant differences in mental health spending (on all mental health care, outpatient mental health care, ED visits with a mental health diagnosis and inpatient admissions with a mental health diagnosis) for 2012 or 2013 SSP entrants.
						Utilization	There were no statistically significant differences in utilization outcomes (on outpatient mental health visits, partial hospitalizations, ED visits with a mental health diagnosis and inpatient admissions with a mental health

© 2020 Carlo AD et al. *JAMA Network Open.*

APM	Payment Model	Intervention	Comparison Group	Study Publication	N	Outcome	Findings and Direction of Effect
							diagnosis) for 2012 or 2013 SSP entrants.
						Patient Dumping/ Gaming/ Adverse Selection	In the 2012 SSP, there was a decrease (-0.5 percentage points, p<0.01) in the prevalence of mental illness diagnoses from a pre-implementation baseline of 7.3%-7.5%, while no statistically significant change was noted in the 2013 SSP cohort. However, when differential changes were present, there was no evidence of a selection "away from" individuals with psychotic disorders compared to those with depression. Differential changes in the demographic composition of the 2012 and 2013 SSP ACOs were negligible.
				Busch et al, 2017[16]	All person-year sample size estimates reported are in the pre-intervention period and are for beneficiaries with mental illness.<br><br>2012 entrant SSP ACO group: 79,993 person-years, control group: 864,672 person-years.<br><br>2013 entrant SSP ACO group: 84,099 person-years, comparison group: 1,189,577 person-years	Processes-of-Care	For ACO versus comparison in the 2013 SSP ACOs (pre- to post-contract), there was no significant change in total days of medication supplied, but any antidepressant use decreased (β=-0.5 percentage points, p<0.05), and, among antidepressant users the proportion of days covered increased (β=0.4 days, p<0.05).
Maine Medicaid Accountable	LAN 3A	Maine's Accountable Community beneficiaries - this program was	Non-ACO beneficiaries	Beil et al, 2019[19]	Total weighted N for treatment and comparison	Processes-of-Care	There was no statistically significant difference in 30-day readmissions per 1,000 discharges. Additionally, the ACO

© 2020 Carlo AD et al. *JAMA Network Open.*

APM	Payment Model	Intervention	Comparison Group	Study Publication	N	Outcome	Findings and Direction of Effect
Communities Initiative		evaluated on the total cost of care for physical and behavioral health services. The AC one-sided risk program (organizations share in savings, but not losses) allowed for up to 50 percent of shared savings (capped at a maximum savings of 10 percent of benchmark total cost of care), with no downside risk (organizations share in losses). Performance on 17 quality metrics proportionately affected the amount of savings[17,18]			group - 156,313 (24,976 for readmissions outcome, 10,677 for antidepressant adherence outcomes and 4,591 for hospitalization discharge follow-up outcomes)		was not associated with any changes in the percentage of patients remaining on antidepressant medication for at least 84 or 180 days. Finally, the ACO was not associated with any change in the percentage of patients following up within 7 or 30 days of discharge from hospitalization for mental illness.
						Spending	The ACO was not associated with significant differences in total per-member-per-month expenditures.
						Utilization	The ACO was not associated with any significant difference in all-cause inpatient admissions or ED visits per 1,000 beneficiaries.
Vermont Medicaid Shared Savings Program (VMSSP)	LAN 3A	Vermont Medicaid Shared Savings Program beneficiaries - developed using components of the Medicare Shared Savings Program (SSP). Vermont incrementally phased covered services into its ACO program, with behavioral health not being an optional covered service until year 2. In Track 1, the ACO is not exposed to downside risk (organizations share in losses) but has an opportunity for shared savings. Shared savings calculations are based on a core set of 28 measures[18,20].	Non-ACO beneficiaries	Beil et al, 2019[19]	Total weighted N for treatment and comparison group - 237,699 (19,975 for readmissions outcome and 6,292 for hospitalization discharge follow-up outcomes)	Processes-of-Care	There was no statistically significant difference in 30-day readmissions per 1,000 discharges. Additionally, the ACO was not associated with any changes in the percentage of patients following up within 7 or 30 days of discharge from hospitalization for mental illness.
						Spending	The ACO was associated with decreases in total per-member-per-month expenditures (β=-$61.77, 90%CI -87.18 to -36.36, p<0.001).
						Utilization	The ACO was associated with differences in all-cause inpatient admissions per 1,000 beneficiaries (β=-10.9, 90%CI -15.1 to -6.7, p<0.001) and ED visits per 1,000 beneficiaries (β=-27.0, 90%CI -34.0 to -20.1, p<0.001).
Medicare Pioneer Accountable Care	LAN 3B	ACO beneficiaries - The Pioneer ACO included 5 possible tracks, with 4 including 2-sided risk	Non-ACO beneficiaries	Busch et al, 2016[15]	All person-year sample size estimates reported are in	Processes-of-Care	For 2012 and 2013 (Post-years 1 and 2) Pioneer ACO, none of the process-of-care outcomes (30-day mental health readmissions, outpatient mental health

© 2020 Carlo AD et al. *JAMA Network Open.*

APM	Payment Model	Intervention	Comparison Group	Study Publication	N	Outcome	Findings and Direction of Effect
Organizations (ACOs)		(organizations share in savings and losses) in the first year and all 5 including 2-sided risk in subsequent years. Sharing savings and financial risk details differed across tracks and from year to year. In the first 2 years across tracks, first-dollar shared savings and losses (i.e., applies to an ACO's total savings or losses above or below its benchmark) ranged from 50-70% of Medicare Parts A and B revenue, with loss sharing limits ranging from 5-15%. The threshold for shared savings, the minimum savings rate across tracks ranged from 1-2.7%. In year 3, for ACOs meeting certain benchmarks, all five tracks had options for population-based (i.e., non-fee-for-service) payment of 0-100% of expected Medicare Parts A and B revenue[21].			the pre-intervention period and are for beneficiaries with mental illness.<br><br>Pioneer ACO group: 38,517-person-years; control group: 864,672 person-years		follow-up within 7 days of discharge, or identified as having a depressive disorder) were statistically significant.
						Clinical Outcomes	There is no evidence that the Pioneer ACO affected mental health status based on Medicare beneficiary self-report (measured by the Consumer Assessment of Healthcare Providers and Systems)
						Spending	Beneficiaries treated under Pioneer ACO contracts had an average reduction of $170 (p<0.05) in total mental health spending in 2012, largely attributed to reductions in inpatient spending, and $5 per beneficiary reduction in ED spending (p<0.05). There was no significant change in total mental health care or inpatient spending in 2013
						Utilization	In 2012, there was no significant change in the number of inpatient hospitalizations with a mental health diagnosis. Additionally, there were no changes in the per-beneficiary counts of outpatient mental health visits, partial hospitalization stays or ED visits in 2012. There were no changes in any utilization measures (outpatient mental health visits, partial hospitalizations, ED visits with a mental health diagnosis or inpatient admissions with a mental health diagnosis) in 2013.
						Patient Dumping/ Gaming/ Adverse Selection	The proportion of attributed beneficiaries with a mental illness diagnosis in the claims data decreased from a pre-implementation baseline of 7.5% in 2012 (β=-0.2%, p<0.05) and in 2013 (β=-0.5%, p<0.001) relative to the control group. Among those with a mental illness, differential changes in the sociodemographic and clinical characteristics of ACO-attributed beneficiaries (relative to non-ACO

© 2020 Carlo AD et al. *JAMA Network Open.*

APM	Payment Model	Intervention	Comparison Group	Study Publication	N	Outcome	Findings and Direction of Effect
							controls) in the Pioneer program were minimal in 2012; in 2013 there was a differential change in the percentages of dually eligible (β=-1.1, p<0.05) and disabled beneficiaries (β=-1.5, p<0.01).
				Busch et al, 2017[16]	All person-year sample size estimates reported are in the pre-intervention period and are for beneficiaries with mental illness.<br><br>Pioneer ACO group: 38,517-person-years; control group: 864,672 person-years	Processes-of-Care	For ACO vs. comparison in the Pioneer ACOs (pre- to post-contract) in post-year 1 (i.e., 2012), days of medication supplied increased (β=4.2 days, p<0.01). Over the same period, there was no change in any antidepressant use, but the proportion of days covered by days supplied (among antidepressant users) increased (β=0.8 percentage points, p<0.01). In the 2013 Pioneer ACO (pre- to post-contract), the proportion of days covered by days supplied (among antidepressant users) significantly increased (β=0.9 percentage points, p<0.05).
Minnesota Integrated Health Partnerships Program	LAN 3B	Minnesota's Integrated Health Partnership organizations - these two-sided risk (share in savings and losses) delivery systems provide outpatient and inpatient care, with each serving more than 2,000 members. Risk was incrementally phased-in over three-years, with downside risk first appearing in year 2. Both the state and its managed care organizations pay portions of shared savings to IHPs or share in losses from IHPs that do not achieve savings. The IHPs report on 32 quality metrics[17,18]	Non-ACO beneficiaries	Beil et al, 2019[19]	Total weighted N for treatment and comparison group - 876,307 (149,830 for readmissions outcome, 96,944 for antidepressant adherence outcomes and 710,275 for the per beneficiary per month expenditures outcome)	Processes-of-Care	There was no statistically significant difference in 30-day readmissions per 1,000 discharges. Additionally, the ACO was not associated with any change in the percentage of patients remaining on antidepressant medication for at least 84 days but was associated with a decrease in the percentage remaining on antidepressants after ≥180 days (β=-1.4 percentage points, 90%CI -2.2 to -0.7, p<0.002).
						Spending	The ACO was not associated with significant differences in total per-member-per-month expenditures.
						Utilization	The ACO was associated with an increase in all-cause inpatient admissions per 1,000 beneficiaries (β=3.6, 90%CI 1.6 to 5.7, p=0.003) and a decrease in ED visits per 1,000 beneficiaries (β=-22.8, 90%CI -25.6 to -19.9, p<0.001).

© 2020 Carlo AD et al. *JAMA Network Open.*

APM	Payment Model	Intervention	Comparison Group	Study Publication	N	Outcome	Findings and Direction of Effect
Delaware division of Substance Abuse and Mental Health (DSAMH) - Outpatient Services APM (2002)	LAN 4A	Value-based payment for outpatient substance use treatment in Delaware - three measurable patient behaviors were selected as performance criteria - (1) Capacity Utilization - the DSAMH paid 1/12 of the total annual operating costs for a program at the end of each month contingent upon the program maintaining at least an 80% rate of their utilization capacity - utilization rates less than 80% led to incrementally reduced reimbursement (to a minimum of 50% reimbursement for utilization rates below 60%); (2) Active Participation in Treatment - the DSAMH would pay an additional 1% to programs for meeting each of 4 specific performance targets related to treatment participation; (3) Program Completion - programs could earn a $100 bonus for each client that completed their addiction program as defined by the DSAMH (up to a pre-defined maximum)	Substance use disorder care in Maryland over the same period	Stewart et al, 2013[22]	Intervention: all adult clients treated in publicly funded outpatient Alcohol and Other Drug (AOD) treatment programs between 1998 and 2006 in Delaware (12,368 patients); Comparison: Patients treated in similar programs over the same period in Maryland (147,151 patients)	Processes-of-Care	The APM was associated with reduced patient average wait times in 2002–2003 (β=-13.27 days, SE 2.36, p<0.0001) and 2004-2006 (β=-20.04 days, SE 2.22, p<0.0001). For length of stay in the outpatient treatment program, the APM was associated with increases in 2002–2003 (β=24.35 days, SE 10.83, p=0.02) and 2004–2006 (β=22.05 days, SE 8.46, p=0.01)
						Patient Dumping/ Gaming/ Adverse Selection	Although the data were not shown in the published article, the authors did report that analyses were conducted for patient dumping, with results showing that the "population did appear to become more severe over time."
			Pre-intervention phase	McLellan et al, 2008[23]	DSAMH contracts with the 5 outpatient treatment provider organizations that operated all 11 outpatient programs in the state. Total annual enrolled patient counts ranged from 1204 to 2227.	Processes-of-Care	Between 2001 and 2006, the average proportion of patients meeting active participation requirements increased from 53% to 70%.
						Utilization	Between 2001 and 2006, average capacity utilization increased from 54% to 95%.
						Patient Dumping/ Gaming/ Adverse Selection	The data generally demonstrate stable patient characteristics pre- and post-intervention. There were three notable exceptions: (1) the percentage of patients endorsing a history of mental illness increased over time from 12% to 23%, (2) the percentage of women attending treatment increased from 20% to 25%, and the percentage of patients working jobs with wages decreased from 57% to 52%.
Delaware division of Substance Abuse and Mental Health	LAN 4A	Value-based payment for substance use treatment in Delaware - the DSAMH contracts with only one vendor/program that	Pre-intervention phase	Haley et al, 2011[24]	All publicly funded patients admitted to a Delaware organization's	Utilization	Vendor/program maintained the requirement for 90% occupancy and accomplished at least 25% entry into outpatient treatment within 7 days following discharge from detoxification.

© 2020 Carlo AD et al. *JAMA Network Open.*

APM	Payment Model	Intervention	Comparison Group	Study Publication	N	Outcome	Findings and Direction of Effect
(DSAMH) - Detoxification Care Transition APM (2008)		offers detoxification and follow-up outpatient services. The APM contract included base and monthly incentive components. There were 3 performance measures in the contract: (1) vendor could earn up to 90% of the monthly base payment if the average daily census of patients was maintained at 90% or higher, (2) vendor could earn the remaining 10% if at least 25% of patients who completed detoxification entered either an outpatient or residential treatment program following discharge from detoxification program, and (3) vendor could earn a $500 payment for each “frequent detoxification” (3 or more prior detoxification visits) patient who entered outpatient care/residential within 7 days of discharge from detoxification unit and remained for a specified period of time (60 days outpatient or 30 days residential).			detoxification unit from July 1, 2005 to June 30, 2006 (1,920 patients) and July 1, 2007 to June 30, 2008 (1,980 patients).		However, only 8% of “frequently detoxing” patients completed detoxification and entered outpatient/residential care for the minimum specified duration. There were no regression analyses examining whether the APM was associated with changes in the outcome measures of interest (i.e., what was contracted for in the APM).
						Patient Dumping/ Gaming/ Adverse Selection	Regression analyses indicated there were some significant differences in the demographic and clinical characteristics of the patients treated in the detoxification unit pre- vs. post-intervention. Compared to the pre-intervention cohort, the post-intervention cohort had significantly greater odds of being White (β=0.265, SE 0.077, p=0.001), homeless (β=0.244, SE 0.084, p=0.03), older (β=0.020, SE 0.004, p<0.001), reporting heroin as primary substance (β=0.311, SE 0.096, p=0.001), and having a greater length of stay in the detoxification unit (β=0.050, SE 0.025, p=0.042). Additionally, the post-intervention cohort had significantly lower odds of being Latino (β=-0.259, SE 0.128, p=0.042), having a history of military service (β=-0.424, SE 0.139, p=0.002), reporting alcohol as primary substance (β=-0.244, SE 0.117, p=0.037), mean age at first use (β=-0.016, SE 0.005, p=0.002), and current legal involvement (β=-0.181, SE 0.080, p=0.024).

© 2020 Carlo AD et al. *JAMA Network Open.*

APM	Payment Model	Intervention	Comparison Group	Study Publication	N	Outcome	Findings and Direction of Effect
Maine Addiction Treatment System (MATS): Phase 1 of performance-based contracting (1992)	LAN 4A	Value-based payment for substance use treatment in Maine - the Office of Substance Abuse (OSA) reviewed annual client outpatient, residential and detoxification performance data as assessed by 24 efficiency, effectiveness and special populations measures. These reviews resulted in 1 of 6 possible outcomes in the following fiscal year - (1) programs could have small reductions in funding (though payments were ultimately not adjusted immediately), (2) programs, if low-performing, could transition to fee-for-service reimbursement, (3) programs could have specific conditions imposed by the OSA (4) contracts with programs could be renewed for 6 months instead of the typical 12 months, (5) programs could be rewarded for "good" performance with additional federal block grant funds, (6) the OSA could encourage programs to "expand their scope." Evaluations	Pre-intervention phase	Commons et al, 1997[25]	877 patients were included in the effectiveness model and 827 patients were included in the efficiency model	Clinical Outcomes	Pre versus post effectiveness outcomes (defined by 15 specific effectiveness-related metrics, where the score reflects the share of these metrics that were met) improved by an average of 0.8% per quarter ($p<0.01$). The regression equation estimates and OSA-APM interaction term ($\beta=0.374$, $p<0.01$, $R^2= 0.270$) suggest that programs that received a higher proportion of their funding from the OSA experienced improved effectiveness relative to those that received a lower proportion of their funding from the OSA. For example, a program with an OSA payment share 20% greater the mean compared to other programs (49% across all programs) would have an estimated effectiveness improvement of 13% (with the average program improving by 5.7%).
						Utilization	Pre versus post outcomes for efficiency (defined as meeting minimum service delivery) improved by an average of 2.2% per quarter ($p<0.01$). The regression equation estimates and OSA-APM interaction term ($\beta=0.374$, $p<0.01$, $R^2= 0.270$) suggest that programs that received a higher proportion of their funding from the OSA experienced improved efficiency. For example, a program with an OSA payment share 20% above the mean (49% across all programs) would have an estimated efficiency improvement of 0.1% (while programs that depended less on the OSA for funding had an average decrease in efficiency by 26.9%).

© 2020 Carlo AD et al. *JAMA Network Open.*

APM	Payment Model	Intervention	Comparison Group	Study Publication	N	Outcome	Findings and Direction of Effect
		occurred annually, making timely feedback unavailable to programs.		Lu 1999[26]	The pre-value-based payment sample consisted of 6,717 patients and the post-value-based payment sample consisted of 6,175 patients	Patient Dumping/ Gaming/ Adverse Selection	After controlling for gaming, the APM was associated with no significant change in improving the effectiveness of the clinical outcomes. The APM did have a significant effect when gaming was ignored (β=0.1076, t-stat=4.8886, p<0.01) and on individual processes-of-care, clinical and utilization outcomes that were measured/incentivized as part of the contract. However, when relapse (a clinical outcome measure excluded from the contract) was included in the model, the true treatment outcome of APM was not statistically significant.
				Lu et al, 2003[27]	The pre-value-based payment sample consisted of 7,777 patients and the post-value-based payment sample consisted of 11,195 patients	Patient Dumping/ Gaming/ Adverse Selection	The APM was associated with improved patient-provider matching for the first (β=0.22, t-stat=6.08, p<0.01), but not second episode of illness. It was also associated with improved patient-provider matching for specific levels of care, such as residential care, based on the APM*residential interaction term estimate for the first (β=1.31, t-stat=5.81, p<0.01) and second (β=1.14, t-stat=4.55, p<0.01) illness episodes. Additionally, the APM was associated with increased likelihood of having deliberate action taken to refer the patient to another substance abuse service at the time of discharge (β=0.10, t-stat=2.20, p<0.05). There was not, however, evidence for association of the APM with patient dumping, defined as a patient being referred from one provider to the next without being treated.
				Lu et al, 2006[28]	The sample included alcohol abuse treatment episodes of 988 patients with data recorded in the Maine Addiction	Patient Dumping/ Gaming/ Adverse Selection	The APM was associated with discrepant accounts in the reporting system to the state versus medical record abstracts, with the reporting system documenting more frequent substance use on admission (β=0.375, z-stat=0.160, p<0.05), and less frequent

© 2020 Carlo AD et al. *JAMA Network Open.*

APM	Payment Model	Intervention	Comparison Group	Study Publication	N	Outcome	Findings and Direction of Effect
					Treatment System (MATS)		use on discharge (β=0.436, z-stat=0.218, p<0.05). After the APM was implemented, clinician report of patient drinking frequency was 9% higher (marginal effect) in the reporting system than in the clinical record on admission and 4% lower (marginal effect) in the reporting system than in the medical record upon discharge.
			Medicaid beneficiaries with no APM exposure	Shen et al, 2003[29]	Intervention: pre-pay-for-performance group of 983 patients and post-pay-for-performance group of 1,384 patients. Comparison: pre-group of 1,191 patients and post- group of 1,994 patients	Patient Dumping/ Gaming/ Adverse Selection	The APM was associated with a decrease in the proportion of patients in outpatient care who were determined to be the most severely ill (β=-.074, 95%CI -0.116 to -0.0317, p<0.01), suggesting that outpatient providers engaged in activities to attract the less severely ill as a result of the APM. The study was unable to evaluate if this resulted in patients being "dumped" (i.e., not receiving treatment) or being appropriately referred to another provider (e.g., another provider could be a better "match" for patient based on patient severity and provider service or level of care offered).
Maine Addiction Treatment System (MATS): Phase 2 of performance-based contracting (2007)	LAN 4A	The second iteration of value-based payment for substance use treatment in Maine - due to mixed results from the first performance-based contracting system as well as concerns about adverse selection and gaming practices, the OSA restructured its performance-based contracting system in 2007. This new system included base payments, incentive payments and penalties and was paid quarterly (instead of	Maine residents treated in clinics with no APM exposure	Brucker et al, 2011[30]	Outpatient intervention: 3,915 patients; Outpatient comparison: 2,745 patients Intensive outpatient intervention: 1,156 patients Intensive outpatient comparison: 1,312 patients	Processes-of-Care	For the outpatient program and the intensive outpatient program, the APM was not associated with any significant changes in time to assessment, time to treatment, or level of patient participation. For the intensive outpatient program, the APM was not associated with any significant change in program completion.
						Utilization	For the outpatient program, the APM was not associated with any significant change in length of stay.
				Stewart et al, 2018[31]	The matched sample (including patients exposed and unexposed	Processes-of-Care	The APM was not associated with any significant change in waiting time for outpatient or intensive outpatient program services.

© 2020 Carlo AD et al. *JAMA Network Open.*

APM	Payment Model	Intervention	Comparison Group	Study Publication	N	Outcome	Findings and Direction of Effect
		annually). Programs that exceeded 100% of contracted units of service per quarter received an incentive payment of 5% of their quarterly payment. At the same time, programs with less than 90% of contracted service units received a 5% cut in payment for that quarter. Additionally, for outpatient and intensive outpatient care, programs' performance on four access and retention measures were assessed quarterly, with these results impacting eligibility for incentive payments, base contract payments, and penalties. In total, programs could gain or lose up to 9% of their contracted payment amount under this contract.			to performance-based contracting) consisted of 26,722 outpatient and 12,210 intensive outpatient program admissions	Patient Dumping/ Gaming/ Adverse Selection	In the outpatient program and intensive outpatient program, the APM was not associated with any significant changes in the proportion of clients with a history of mental health conditions or level of substance use severity.
BCBSMA (BC/BS of Massachusetts) Alternative Quality Contract (AQC)	LAN 4B	Massachusetts Alternative Quality Contract (AQC) beneficiaries - the AQC paid participating organizations via a risk-adjusted, prospective payment (i.e., non-fee-for-service) for all primary and specialty care provided to a population for a five- year period. AQC organizations were eligible for bonuses, in the initial years of up to 10% of their budget and later	Non-AQC Beneficiaries	Barry et al, 2015[32]	Intervention: Total AQC (533,568 person-years), exposed to BH risk (236,542 person-years), and unexposed to BH risk (297,026 person-years); Comparison: Total (2,999,221 person-years)	Processes-of-Care	The AQC did not have a significant effect on the average number of inpatient mental health days conditional on inpatient mental health use. AQC beneficiaries in no-risk organizations had a higher average number of outpatient mental health visits conditional on outpatient mental health use (β=0.51 visits, 95%CI 0.04 to 0.95, p<0.05), while there was no significant effect overall or in behavioral health risk organizations. AQC beneficiaries had a higher average number of medication management visits conditional on outpatient mental health use overall (β=0.08 visits, 95%CI 0.03 to 0.13,

© 2020 Carlo AD et al. *JAMA Network Open.*

APM	Payment Model	Intervention	Comparison Group	Study Publication	N	Outcome	Findings and Direction of Effect
		as a per member per month amount, based on performance on 64 outpatient and hospital measures. A subset of initial 5-year AQC contracts (5/12) included risk for MH/SUD specialty service use[32]					p<0.01) and in organizations facing behavioral health risk (β=0.09 visits, 95%CI 0.02 to 0.17, p<0.05). The average number of psychotherapy visits conditional on outpatient mental health use increased for AQC beneficiaries in no-risk organizations (β=0.48 visits, 95%CI 0.02 to 0.91, p<0.05), but not overall. The average number of thirty-day-equivalent psychotropic medication prescriptions conditional on psychotropic medication use increased overall (β=0.23, 95%CI 0.03 to 0.42, p<0.01) and in no-risk organizations (β=0.34, 95%CI 0.09 to 0.58, p<0.01), but not in organizations accepting behavioral health risk.
						Spending	Significant decreases in average total health care spending conditional on mental health use were noted for the total AQC beneficiary group relative to comparison (β=-$189, 95%CI -368 to -9, p<0.05) and for beneficiaries treated in organizations facing behavioral health risk (β=-$238, 95%CI -468 to -9, p<0.05), but not for beneficiaries in no-risk organizations. The AQC did not have significant effects on mental health spending conditional on mental health use.
						Utilization	There was a small overall decrease in the probability of using mental health services (β=−1.41percentage points, CI -2.06 to -0.76, p<0.01) among AQC beneficiaries relative to the comparison group. This finding was largely attributable to beneficiaries from organizations facing behavioral health risk (β=−2.09 percentage points, CI -3.29 to -0.99, p<0.01), with no-risk organizations showing no statistically significant difference.

© 2020 Carlo AD et al. *JAMA Network Open.*

APM	Payment Model	Intervention	Comparison Group	Study Publication	N	Outcome	Findings and Direction of Effect
				Stuart et al, 2017[33]	Intervention: Total AQC (10,817 person-years), exposed to BH risk (4,608 person-years), and unexposed to BH risk (6,209 person-years); Comparison group - 50,576 person-years	Processes-of-Care	In AQC provider organizations with contractual downside behavioral health risks, the AQC had no effect on SUD performance measures (HEDIS measures of SUD identification, initiation, or engagement). In AQC no-risk organizations, the AQC was associated with slightly higher rates of identification (β=0.15%, 95%CI 0.04 to 0.26, p=0.007), lower rates of treatment initiation (β=-3.34%, 95%CI -5.68 to -1.00, p=0.005), and lower probability of any SUD medication use conditional on being a SUD service user (β=-2.30%, 95%CI -4.43% to -0.18%, p=0.03).
						Spending	Among organizations that were and were not at-risk for behavioral health, the AQC had no significant effect on average SUD services spending or average total health care spending among SUD service users. Additionally, no significant differences in spending were noted for the AQC overall (regardless of behavioral health risk acceptance).
						Utilization	In the AQC group without behavioral health risk, there was a small increase in the probability of any SUD service use (β=0.16%, 95%CI 0.06 to 0.26, p=0.003). However, in AQC organizations overall and in those with behavioral health risk, there was no significant difference in SUD service use. Among the specific utilization measures, the AQC no-risk group had a lower probability of any substance use disorder medication use conditional on being a substance use disorder service user (β=-2.30%, 95%CI -4.43 to -0.18, p=0.03). All other specific SUD utilization measures were statistically insignificant for organizations with

© 2020 Carlo AD et al. *JAMA Network Open.*

APM	Payment Model	Intervention	Comparison Group	Study Publication	N	Outcome	Findings and Direction of Effect
							behavioral health risk, those with no risk, and overall.
				Donohue et al, 2018[34]	Intervention: opioid use disorders (2,534 person-years) and alcohol use disorders (6,422 person-years); Comparison: opioid use disorders (10,193 person-years) and alcohol use disorders (30,691 person-years)	Spending	There were no significant differences in spending on medication treatment for alcohol or opioid use disorders attributable to the AQC.
						Utilization	There were no significant differences in the use of medication treatment among enrollees with opiate or alcohol use disorders attributable to the AQC. Additionally, there were no significant differences in the number of 30-day prescriptions for addiction medication among enrollees with opiate or alcohol use disorders attributable to the AQC.
Oregon Coordinated Care Organizations (CCOs)	LAN 4B	Oregon Coordinated Care Organizations receive a prospectively paid, global budget (i.e., non-fee-for-service) for all services provided (including physical health, mental health and dental care) that grows at a fixed rate. Additionally, 3% of monthly payments made to the APM are held by the Oregon Health Authority for placement into a quality pool. These funds are distributed annually as incentive payments to the CCOs based on their performance on 17 quality metrics. To receive a full incentive payment, a CCO must: (1) meet benchmarks or improvement targets on at least 12/17 quality metrics and (2) have at least 60%	Pre-intervention phase	Rieckmann et al, 2018[35]	After exclusions, there was a total study CCO population of 516,708	Processes-of-Care	From the pre-intervention to the post-intervention period, screening and brief intervention screening rates went from consistently less than 0.1% of patients to as high as 4.6% of patients after implementation of the intervention. Despite the increase in SBI screening, data did not substantially demonstrate changes in the rates of alcohol or drug use disorder diagnoses. Results also showed that screening was not associated with initiation of alcohol or drug use treatment at 6 or 12 months.

© 2020 Carlo AD et al. *JAMA Network Open.*

APM	Payment Model	Intervention	Comparison Group	Study Publication	N	Outcome	Findings and Direction of Effect
		of its members enrolled in a patient-centered primary care home.					

Note: Abbreviations: LAN: Health Care Payment Learning and Action Network; APM: alternative payment model; RR: relative risk; OR: odds ratio; CI: confidence interval; MH: mental health; SUD: substance use disorder; BH: behavioral health; PHQ-9: patient health questionnaire-9; HR: hazard ratio; A-CRA: Adolescent Community Reinforcement Approach; SD: standard deviation; ACO: accountable care organization; ED: emergency department; SSP: Medicare Shared Savings Program; AQC: alternative quality contract

© 2020 Carlo AD et al. *JAMA Network Open.*

eReferences.

1. Bailitt Health. Categorizing Value-Based Payment Models According to the LAN Alternative Payment Model Framework: Examples of Payment Models by Category. 2018;(February):1-13. http://shvs.org/wp-content/uploads/2018/02/SHVS_APM-Categorization_Brief-Final.pdf.
2. Health Care Payment Learning & Action Network. https://hcp-lan.org. Published 2018. Accessed September 20, 2018.
3. Ross KM, Gilchrist EC, Melek SP, Gordon PD, Ruland SL, Miller BF. Cost savings associated with an alternative payment model for integrating behavioral health in primary care. *Transl Behav Med*. 2018;(May). doi:10.1093/tbm/iby054
4. Garner BR, Godley SH, Bair CML. The impact of pay-for-performance on therapists' intentions to deliver high-quality treatment. *J Subst Abuse Treat*. 2011;41(1):97-103. doi:10.1016/j.jsat.2011.01.012
5. Garner BR, Godley SH, Dennis ML, Hunter BD, Bair CM, Godley MD. Using pay for performance to improve treatment implementation for adolescent substance use disorders: results from a cluster randomized trial. *Arch Pediatr Adolesc Med*. 2012;166(10):938-944. doi:10.1001/archpediatrics.2012.802
6. Garner BR, Lwin AK, Strickler GK, Hunter BD, Shepard DS. Pay-for-performance as a cost-effective implementation strategy: results from a cluster randomized trial. *Implement Sci*. 2018;13(1):92. doi:10.1186/s13012-018-0774-1
7. Lee MT, Garnick DW, O'Brien PL, et al. Adolescent treatment initiation and engagement in an evidence-based practice initiative. *J Subst Abuse Treat*. 2012;42(4):346-355. doi:10.1016/j.jsat.2011.09.005
8. Shepard DS, Calabro JA, Love CT, McKay JR, Tetreault J, Yeom HS. Counselor incentives to improve client retention in an outpatient substance abuse aftercare program. *Adm Policy Ment Health*. 2006;33(6):629-635. doi:10.1007/s10488-006-0054-2
9. Vandrey R, Stitzer ML, Acquavita SP, Quinn-Stabile P. Pay-for-performance in a community substance abuse clinic. *J Subst Abuse Treat*. 2011;41(2):193-200. doi:10.1016/j.jsat.2011.03.001
10. Unützer J, Chan YF, Hafer E, et al. Quality improvement with pay-for-performance incentives in integrated behavioral health care. *Am J Public Health*. 2012;102(6):E41-E45. doi:10.2105/AJPH.2011.300555
11. Bao Y, McGuire TG, Chan Y-F, et al. Value-based payment in implementing evidence-based care: the Mental Health Integration Program in Washington state. *Am J Manag Care*. 2017;23(1):48-53.
12. Schmutte T, Van der Heide L, Szczygiel L, Phelan A, Davidson L, Plant R. A Pay-for-Performance Initiative to Reduce Pediatric Psychiatric Inpatient Length of Stay. *Psychiatr Serv*. 2019;70(2):156-158. doi:10.1176/appi.ps.201800190
13. American Academy of Family Physicians (AAFP). Medicare shared savings program: Accountable care organizations final rule. https://www.aafp.org/dam/AAFP/documents/advocacy/payment/medicare/ES-ACO-Rule-110211.pdf. Published 2011. Accessed September 24, 2019.
14. Berwick DM. Making Good on ACOs' Promise — The Final Rule for the Medicare Shared Savings Program. *N Engl J Med*. 2011;365(19):1753-1756. doi:10.1056/NEJMp1111671

15. Busch AB, Huskamp HA, McWilliams JM. Early efforts by medicare accountable care organizations have limited effect on mental illness care and management. *Health Aff*. 2016;35(7):1247-1256. doi:10.1377/hlthaff.2015.1669
16. Busch AB, Huskamp HA, Kreider AR, McWilliams JM. Medicare accountable care organizations and antidepressant use by patients with depression. *Psychiatr Serv*. 2017;68(11):1193-1196. doi:10.1176/appi.ps.201600538
17. Lloyd J, Houston R, Mcginnis T. *Medicaid Accountable Care Organization Programs: State Profiles*.; 2015. http://www.chcs.org/media/Medicaid-Accountable-Care-Organization-Programs-State-Profiles.pdf.
18. RTI International. *State Innovation Models (SIM) Initiative Evaluation - Model Test Year Five Annual Report*.; 2018. https://downloads.cms.gov/files/cmmi/sim-rd1-mt-fifthannrpt.pdf.
19. Beil H, Feinberg RK, Patel S V, Romaire MA. Behavioral Health Integration With Primary Care: Implementation Experience and Impacts From the State Innovation Model Round 1 States. *Milbank Q*. 2019. doi:10.1111/1468-0009.12379
20. Rutledge RI, Romaire MA, Hersey CL, Parish WJ, Kissam SM, Lloyd JT. Medicaid Accountable Care Organizations in Four States: Implementation and Early Impacts. *Milbank Q*. 2019;97(2):583-619. doi:10.1111/1468-0009.12386
21. Center for Medicare & Medicaid Innovation (CMMI). *Alternative Payment Arrangements for the Pioneer ACO Model Background*.; 2011. https://innovation.cms.gov/Files/x/Pioneer-ACO-Model-Alternative-Payment-Arrangements-document.pdf.
22. Stewart MT, Horgan CM, Garnick DW, Ritter G, McLellan AT. Performance contracting and quality improvement in outpatient treatment: effects on waiting time and length of stay. *J Subst Abus Treat*. 2013;44(1):27-33. doi:10.1016/j.jsat.2012.02.001
23. McLellan AT, Kemp J, Brooks A, Carise D. Improving public addiction treatment through performance contracting: the Delaware experiment. *Health Policy*. 2008;87(3):296-308. doi:10.1016/j.healthpol.2008.01.010
24. Haley SJ, Dugosh KL, Lynch KG. Performance contracting to engage detoxification-only patients into continued rehabilitation. *J Subst Abuse Treat*. 2011;40(2):123-131. doi:10.1016/j.jsat.2010.09.001
25. Commons M, McGuire TG, Riordan MH. Performance contracting for substance abuse treatment. *Heal Serv Res*. 1997;32(5):631-650.
26. Lu M. Separating the True Effect from Gaming in Incentive-Based Contracts in Health Care. *J Econ Manag Strateg*. 1999;8(3):383-431. doi:10.1111/j.1430-9134.1999.00383.x
27. Lu M, Ma CA, Yuan L. Risk selection and matching in performance-based contracting. *Health Econ*. 2003;12(5):339-354. doi:10.1002/hec.734
28. Lu M, Ma CA. Financial incentives and gaming in alcohol treatment. *Inquiry*. 2006;43(1):34-53. doi:10.5034/inquiryjrnl_43.1.34
29. Shen Y. Selection incentives in a performance-based contracting system. *Heal Serv Res*. 2003;38(2):535-552.
30. Brucker DL, Stewart M. Performance-based contracting within a state substance abuse treatment system: a preliminary exploration of differences in client access and client outcomes. *J Behav Heal Serv Res*. 2011;38(3):383-397. doi:10.1007/s11414-010-9228-5
31. Stewart MT, Reif S, Dana B, et al. Incentives in a public addiction treatment system: Effects on waiting time and selection. *J Subst Abuse Treat*. 2018;95:1-8.

© 2020 Carlo AD et al. *JAMA Network Open.*

doi:10.1016/j.jsat.2018.09.002
32. Barry CL, Stuart EA, Donohue JM, et al. The early impact of the "Alternative Quality Contract" On mental health service use and spending in massachusetts. *Health Aff*. 2015;34(12):2077-2085. doi:10.1377/hlthaff.2015.0685
33. Stuart EA, Barry CL, Donohue JM, et al. Effects of accountable care and payment reform on substance use disorder treatment: evidence from the initial 3 years of the alternative quality contract. *Addiction*. 2017;112(1):124-133. doi:10.1111/add.13555 10.1111/add.13555. Epub 2016 Sep 24.
34. Donohue JM, Barry CL, Stuart EA, et al. Effects of Global Payment and Accountable Care on Medication Treatment for Alcohol and Opioid Use Disorders. *J Addict Med*. 2018;12(1):11-18. doi:10.1097/ADM.0000000000000368
35. Rieckmann T, Renfro S, McCarty D, Baker R, McConnell KJ. Quality Metrics and Systems Transformation: Are We Advancing Alcohol and Drug Screening in Primary Care? *Health Serv Res*. 2018;53(3):1702-1726. doi:10.1111/1475-6773.12716

© 2020 Carlo AD et al. *JAMA Network Open.*
